# Is the transition from primary to secondary school a risk factor for energy balance-related behaviours? A systematic review

**DOI:** 10.1017/S1368980023000812

**Published:** 2023-09

**Authors:** Helga Emke, Coosje Dijkstra, Stef Kremers, Mai JM Chinapaw, Teatske Altenburg

**Affiliations:** 1 Vrije Universiteit Amsterdam, Department of Health Sciences, Faculty of Science, De Boelelaan 1117 Amsterdam, The Netherlands; 2 Amsterdam Public Health Research Institute, Health Behaviour and Chronic Diseases and Methodology, Amsterdam, The Netherlands; 3 Maastricht University Medical Centre, Department of Health Promotion, NUTRIM School of Nutrition and Translational Research in Metabolism, P. Debyelaan 25, Maastricht, The Netherlands; 4 Amsterdam UMC location Vrije Universiteit Amsterdam, Public and Occupational Health, De Boelelaan 1117, Amsterdam, The Netherlands

**Keywords:** Adolescents, Obesity, Physical activity, Sedentary behaviour, Sleep behaviour, Dietary behaviour

## Abstract

**Objective:**

The substantial changes in the physical and social environment during the transition from primary to secondary school may significantly impact adolescents’ energy balance-related behaviours (i.e. dietary behaviour, sedentary behaviour, sleep behaviour and physical activity (PA)). This is the first review systematically summarising evidence on changes in four energy balance-related behaviours of adolescents across the school transition from primary to secondary school.

**Design:**

For this systematic review, the electronic databases Embase, PsycINFO and SPORTDiscus were searched for relevant studies from inception to August 2021. PubMed was searched for relevant studies from inception to September 2022. Inclusion criteria were: (i) longitudinal studies reporting; (ii) one or more energy balance-related behaviours; and (iii) across the school transition, that is, with measurement(s) during both primary and secondary school.

**Setting:**

Transition from primary to secondary school

**Participants:**

Adolescents across the transition from primary to secondary school.

**Results:**

Thirty-four studies were eligible. We found strong evidence for an increase in sedentary time, moderate evidence for a decrease in fruit and vegetable consumption, and inconclusive evidence for a change in total, light, and moderate-to-vigorous PA, active transport, screen time, unhealthy snack consumption, and sugar-sweetened beverages consumption among adolescents across the school transition.

**Conclusions:**

During the transition from primary to secondary school, sedentary time and fruit and vegetable consumption tend to change unfavourably. More high-quality, longitudinal research is needed specifically on changes in energy balance-related behaviour across the school transition, especially regarding sleep behaviour. (Prospero registration: CRD42018084799)

The number of adolescents with overweight and obesity is growing worldwide, and this public health problem is currently one of the most serious challenges of the twenty-first century^(1)^. Adolescents with overweight and obesity are at increased risk of various lifestyle-related diseases later in life, including hypertension, hypercholesterolemia, diabetes mellitus type 2 and CVD^(2–4)^. Additionally, due to stigmatisation, adolescents with overweight and obesity tend to have lower self-esteem^(5)^, which can result in loneliness, sadness and tenseness^(6)^. It is therefore important to prevent overweight and obesity during childhood. Childhood overweight and obesity are caused by different behaviours that interact and influence each other^(7)^, including an unhealthy diet, reduced sleep duration, low levels of physical activity (PA) and excessive screen time^(8–10)^. During adolescence, obesity prevalence is higher among 12–19-year-olds than 4–11-year-olds^(11,12)^. The transition from primary to secondary school might contribute to this increase in obesity prevalence.

Previous reviews on the age period of the transition showed that adolescents’ PA levels decreased, while their sedentary behaviour (SB) and screen time increased^(13,14)^. Other studies in the UK and the USA showed that dietary patterns from adolescents in secondary schools are more unfavourable (i.e. an increase in sugar-sweetened beverages (SSB) intake and a decrease in fruit and vegetable intake)^(15–17)^. In addition, when adolescents grow older they tend to increase their screen time^(18)^, which is unfavourable since a systematic review showed that screen time was associated with reduced sleep duration and increased sleep problems among adolescents^(10)^.

There are several explanations for the change towards unfavourable energy balance-related behaviours when adolescents transition from primary to secondary school. For example, when adolescents grow older changes in the biological regulatory processes occur that are known to cause a biological delay in the timing of sleep onset^(19)^. Additionally, parents generally set less rules regarding, for example, screen time when their children grow older^(20)^. The transition in school environment also results in changes in intrapersonal factors and social and physical environmental factors^(21–24)^, including changes in sports facilities, academic expectations and self-judgement of PA skills^(23,24)^. Furthermore, adolescents experience more freedom and receive more pocket money that both enables them to buy high-energy foods and drinks^(25–27)^. On top of that, this period is associated with an increase in travel duration and adolescents experiencing social stress due to the school transition^(28,29)^.

Currently, no systematic review studied dietary behaviour across the transition from primary to secondary school. Furthermore, no recent systematic review examined PA, SB, sleep behaviour and dietary behaviour during the school transition. A combined review is of interest because these behaviours are connected and influence each other, for example, more screen use leads to more unhealthy snacking, less PA and lower sleep quality^(30)^. Therefore, this systematically review summarised the evidence on changes in four energy balance-related behaviours (i.e. PA, SB, sleep behaviour and dietary behaviour) of adolescents in the transition from primary to secondary school.

## Methods

This systematic review was conducted following the PRISMA statement for reporting systematic reviews^(31)^. The review protocol is registered in the International Prospective Register for Systematic Reviews (registration number CRD42018084799 at www.crd.york.ac.uk/prospero/).

### Search strategy

The search strategy included terms related to PA, SB, sleep behaviour, and dietary behaviour and the transition from primary to secondary school. We searched for relevant studies in four electronic databases (PubMed, Embase, PsycINFO and SPORTDiscus) from inception until August 2021. In addition, we manually searched the reference lists of included studies for relevant studies.

### Inclusion criteria

Studies were included if they had a longitudinal design and examined one or more energy balance-related behaviours across the transition from primary/elementary school (hereafter referred to as primary school) to secondary/middle school (hereafter referred to as secondary school), with at least one measurement in adolescents attending the final grades of primary school and one in the same adolescents attending the first grades of secondary school. Only full-text studies published in English in peer-reviewed journals were included.

### Identification of relevant studies

First, one author (HE) performed the search in co-operation with a search specialist from the library of the Vrije Universiteit Amsterdam. Second, two authors independently checked potentially relevant studies by screening the titles and abstracts (HE and CD/TA); when abstracts were not available the studies were included for full-text screening. Third, two authors (HE and CD/TA) independently screened full-text studies to determine whether the inclusion criteria were met. Any discrepancies between the authors were resolved through discussion. A third reviewer (TA/CD) was consulted when consensus could not be reached.

### Data extraction

Two authors (HE and CD/TA) independently extracted data from all included studies, using a structured data extraction form. Information was extracted regarding participant characteristics (i.e. ethnicity and gender), study characteristics (i.e. type of energy balance-related behaviour, length of follow-up and measurement of energy balance-related behaviours) and the study results. To reach consensus for a uniform data extraction procedure, two authors (HE and CD/TA) independently extracted data from the first three studies, before continuing with all other included studies. Discrepancies were resolved through discussion. A third reviewer (TA/CD) was consulted when consensus could not be reached.

### Quality assessment

To assess the methodological quality of the included studies, we used the fourteen-item National Institute of Health (NIH) quality assessment tool for Observational Cohort and Cross-Sectional Studies^(32)^. We included the following quality items: having a clearly stated research question, a clearly specified study population, a representative sample, non-biased recruitment of subjects, justification of sample size, valid and reliable assessment tool, an adequate follow-up rate, and statistical analysis adjusted for potential confounders (for the details see Table 1). Three of the included items were informative, and only the five validity/precision items were included in the quality score^(33)^. Six quality items of the tool were not applicable for our research question and study design and were therefore excluded, including exposure of interest, sufficient time frame, different levels of exposure, exposure measures and assessment, and blinding for exposure outcomes.

**Table 1:** Included quality items from NIH quality assessment tool for observational cohort and cross-sectional studies

Criteria	Rating	I, V/P*
Research question	Studies were rated positively when the research question or objective was clearly stated.	I
Study population	Studies were rated positively when the study population was clearly specified and defined.	I
Representative sample	Studies were rated positively when the target population was adequately represented and more than 50 % of the eligible persons participated in the study.	V/P
Recruitment of subjects	Studies were rated positively when participants were selected or recruited from the same or similar populations (including the same time period) and when inclusion and exclusion criteria were pre-specified and applied uniformly to all participants.	V/P
Sample size	Studies were rated positively when the sample size justification, power calculation, or variance and effect estimates were provided.	I
Outcome measures	Studies were rated positively when using an assessment tool with acceptable^1^ validity and reliability.	V
Follow-up rate	Studies were rated positively when the follow-up rate was at least 70 %.	V
Statistical analysis	Studies were rated positively when the analyses adjusted for key potential confounding variables (e.g. baseline and gender).	V/P

* I, informative criterion; V/P, validity/precision criteria.

^1^Acceptable validity or reliability included that 75 % of the extracted items had a Cronbach’s alpha of above 0·7, and no items were below 0·4.

Quality items were scored following a ‘yes’, ‘no’, ‘cannot be determined’, ‘not applicable’ or ‘not reported’ answering format. Two assessors (HE and TA/CD) independently assessed the quality items of the included studies. Discrepancies were resolved through discussion. A third reviewer was consulted when consensus could not be reached (CD/TA). Studies that included multiple energy balance-related behaviours, used multiple outcomes for one behaviour, for example, moderate-to-vigorous physical activity (MVPA) and active transport or used multiple measurement tools for one behaviour (e.g. objective and self-reported) received multiple scores. A study was considered ‘*strong*’ when scoring 80–100 % of the validity/precision criteria points, ‘*moderate*’ when scoring 40–79 % of the validity/precision criteria points and ‘*poor*’ when scoring 0–39 % of the validity/precision points.

### Best evidence synthesis

We applied best evidence synthesis to draw conclusions regarding the evidence for a change in energy balance-related behaviours across the transition from primary to secondary school. This evidence synthesis is in line with previous reviews^(33–35)^, taking the number of studies, the methodological quality of the studies and the consistency of the findings into account. The level of evidence was defined as:

- Strong evidence: consistent findings in more than two strong quality studies.
- Moderate evidence: consistent findings in one study of strong methodological quality and at least one study of moderate methodological quality or consistent findings in two or more studies of moderate methodological quality.
- Inconclusive evidence: only one study available, or inconsistent findings in two or more studies.

We considered the results within a study consistent when at least 75 % of the outcomes (e.g. total physical activity (TPA), MVPA or transport) within the same behaviour (e.g. PA) showed statistically significant (*P* < 0·05) results in the same direction. Publications based on the same data were only counted once in the best evidence synthesis, that is, combining the results from those publications. When studies described changes in energy balance-related behaviours without testing whether these changes were statistically significant, we contacted the authors by email and requested additional analyses or the necessary data to conduct the analyses ourselves. We contacted the authors of seven studies of which two provided the requested information or dataset. These results of these studies were included in the evidence synthesis^(36,37)^, and the other studies could not be included. We considered the results across studies consistent when at least 75 % of the studies showed results in the same direction, which was defined by significance (*P* < 0·05). Studies with a poor quality rating are included in Table 2 but were not included in the evidence synthesis.

**Table 2:** Study characteristics – sorted by energy balance-related behaviour, study name, quality score and alphabetically by first author

Author (year)	Country/study name	Participants	Energy balance-related behaviour	Length of follow-up	Outcome measurements	Results	Quality assessment
PA							
Clennin *et al.* ^(49)^	USA/TRACK study	*n* 660; 10·6 ± 0·5 years; 54 % girls; 38·3 % non-Hispanic White, 36·1 % non-Hispanic Black, 9·2 % Hispanic, and 16·4 % other	PA	2 years	Accelerometer: PA (min/h)	Decline in PA (β(se)) – 4·5 (0·5) min/h (*P* < 0·001) (Regression models)	100 %
Dowda *et al.* ^(48)^	USA/TRACK study	*n* 555; 10·5 ± 0·5; 56 % girls; 41 % were Black, 36 % White, 8 % Hispanic and 15 % other	PA	1 year	PAQ-C questionnaire, 5-point Likert scale	- Home PA (mean ± sd) decreased from 4·0 ± 3·0 to 2·8 ± 2·3. - Neighbourhood PA (mean ± sd) decreased from 1·8 ± 2·3 to 1·2 ± 1·8. (No statistical analysis)	60 %
Dowda *et al.* ^(50)^	USA/TRACK study	*n* 409; 10·6 ± 0·5 years; 53 % girls; 35 % were Black, 38 % White, 11 % Hispanic and 17 % other	PA	2 years	Accelerometer: MVPA (min/h) Parent reported of child’s PA two items, 5-point Likert scale Child’s self-reported PA four items, 5-point Likert scale	Change in accelerometer-based MVPA (mean ± sd) min/h: 1·6 ± 0·5 to 1·5 ± 0·5 Change in parent report of child’s PA (mean ± sd): 3·1 ± 0·9 to 3·2 ± 1·1 Change in self-reported PA (mean ± sd): 3·3 ± 0·7 to 3·3 ± 0·7 (No statistical analysis)	Parent-reported child’s PA: 40 % Self-reported PA and accelerometer-based MVPA: 60 %
Lau *et al.* ^(43)^	USA/TRACK study	5th grade: *n* 768; 10·6 ± 0·5 years; 54 % girls 6th grade: *n* 751; 50 % girls 7th grade *n* 612; 68 % girls	PA	2 years	Accelerometer: TPA and MVPA during different time periods: school (about 07.45–15.30), afterschool (about 14.25–18.00), evening (about 18.00–22.00) (min/h).	Changes in MVPA and TPA in different time periods (β(se)): - Boys: –0·7 (0·2) (*P* < 0·05) school MVPA, 0·5 (0·34) afterschool MVPA, 0·3 (0·29) evening MVPA, –4·7 (0·6) (*P* < 0·0001) school TPA, –1·4 (0·40) (*P* < 0·05) afterschool TPA, –3·6 (1·1) (*P* < 0·05) evening TPA - Girls: –0·8 (0·1) (*P* < 0·0001) school MVPA, 0·3 (0·1) afterschool MVPA, –0·5 (0·8) (*P* < 0·05) evening MVPA, –7·5 (0·5) (*P* < 0·0001) school TPA, –2·3 (0·3) (*P* < 0·0001) afterschool TPA, –2·9 (0·2) (*P* < 0·0001) evening TPA (Growth curve analysis)	80 %
Pate *et al.* ^(46)^	USA/TRACK study	*n* 828; 10·6 ± 0·5 years in 5th grade; 54 % girls; 38·3 % white, 35·1 % African-American, 9·5 % Hispanic, 17·1 % other.	PA	2 years	Accelerometer: PA (min/h)	Decline in PA (b = –2·9 min/h; 95 CI = –3·1; –2·6) (Growth curve analysis)	80 %
Pate *et al.* ^(47)^	USA/TRACK study	*n* 652; 10·6 ± 0·5 years in 5th grade; 54 % girls; 38·9 % White, 36·2 % Black, 9·2 % Hispanic, 15·7 % other.	PA	2 years	Accelerometer: PA (min/h)	Decline in PA (mean ± sd) from 28·3 ± 4·5 min/h in 5th grade to 24·3 ± 4·5 in 6th grade (No statistical analysis)	40 %
Coombes *et al.* ^(38)^	UK/PEACH project	*n* 518; 10·9 (10·9; 11·0) years; 56 % girls; 60 % non-White (*v*. White)	PA	1 year	Accelerometer: daily counts per minute Questionnaire: mode of travel to/from school	- No differences in PA (34 % of children reported a different mode of travel to school) - Prevalence of walking to school: 79 % at primary school and 58 % at secondary school - Prevalence of cycling to school: just under 4 % at both schools (No statistical analysis)	80 %
Cooper *et al.* ^(28)^	UK/PEACH project	Primary school: *n* 565; 56 % girls Secondary school: *n* 570; 55 % girls	PA	1 year	Accelerometer: weekday MVPA (min/d) Computerised questionnaire: self-reported travel modes to and from school	- Weekday MVPA (mean ± sd): 60·5 ± 22·3 min/d in primary school and 63·4 ± 23·6 in secondary school *P* = 0·017 - Travel mode: walking 77·0 % in primary and 60·7 % in secondary school; cycling 3·2 % in primary and 3·5 % in secondary school (Paired sample *t* tests)	Accelerometer 80 % Questionnaire 60 %
Jago *et al.* ^(41)^	UK/PEACH project	After school MVPA: Boys *n* 371; girls *n* 439 Weekend MVPA: Boys *n* 195; girls *n* 263 Age 10–11 years	PA	1 year	Accelerometer: after school MVPA (between 15.30 and 20.30) and weekend MVPA	- Change in after school MVPA (mean ± sd): –4·6 ± 17·2 min for boys and –2·6 ± 13·1 min for girls; - Change in weekend MVPA (mean ± sd): 4·7 ± 49·0 min for boys and 2·0 ± 29·6 min for girls (No statistical analysis)	60 %
De Meester *et al.* ^(21)^	Belgium	*n* 420; 11·1 ± 0·5 years; 50 % girls; *n* 399 data pedometer steps; *n* 140 accelerometer data	PA	2 years	Accelerometer: total PA in steps/d and total MPVA in min/d Pedometer: weekday step counts Flemish Physical Activity Questionnaire: active travel to/from school (min/d), extracurricular PA (min/d), total PA (min/d)	Standardised β’s (mean ± sd): - Significant increase in active transportation from/to school (b = 5·8 ± 1·2) - Significant decrease in extracurricular PA (b = –10·5 ± 1·3) - Significant decrease in total PA level (b = –8·9 ± 2·6) - No change in accelerometer/pedometer weekday steps (b = –424·0 ± 255·4) - Significant increase in weekday MVPA (b = 4·9 ± 2·4) (Longitudinal multilevel regression analyses)	Accelerometer 80 % Questionnaire 60 %
Garcia *et al.* ^(39)^	USA	*n* 132; 58 % girls; 30·3 % African-American and 69·7 % European American.	PA	1 year	Child/Adolescent Physical Activity Log (self-report; 16 activities)	- No changes in levels of PA (mean ± sd) from 12·8 ± 11·0 to 11·0 ± 9·6 (=spring activity, weighted by intensity) (Student’s *t* test)	0 %†
Harrison *et al.* ^(40)^	UK/SPEEDY study	*n* 299; 10·2 ± 0·3 years; 55 % girls	PA	4 years	Accelerometer: mean minutes of MVPA over different time periods (lunchtime, commuting, school day and after school).	- MVPA (min) declined during lunchtime (mean ± sd): from 14·5 ± 5·8 to 10·9 ± 5·7, school day from 28·2 ± 9·9 to 24·4 ± 10·2 and after school from 28·9 ± 14·2 to 19·8 ± 13·5 All *P* < 0·05 - MVPA (min) increased for time spent commuting (mean ± sd): from 15·9 ± 7·1 to 17·9 ± 11·3 *P* < 0·05 (Wilcoxon rank-sum tests)	40 %
Kirby *et al.* ^(42)^	UK/PASS study	*n* 641; 51 % girls	PA	2–4 years	PAQ-C questionnaire, 5-point Likert scale, children are classified as active with a score of 3 or higher	- Proportion of active pupils decreased over time; 82·1 % at baseline for boys and 61·1 % for girls, 62·9 % for boys at second-year secondary school and 30·5 % for girls, 41·9 % at fourth-year secondary school for boys and 16·6 % for girls. All *P* < 0·001 (chi-square trends)	20 %†
Mikalsen *et al.* ^(52)^	Norway/no study name	7th grade: *n* 306; 12–13 year; 50 % girls 9th grade *n* 160; 14–15 years; 60 % girls	PA	2 years	Accelerometer: MVPA	Two trajectories identified showing a significant decline in MVPA per d/year: Average decline in trajectory-1 (prevalence: *n* 83, 26 % of the total sample): 6·4 min of MVPA/d/year Average decline in trajectory-2 (prevalence: *n* 238, 74 % of the total sample): 14·0 min of MVPA/d/year (Latent growth modelling)	60 %
Remmers *et al.* ^(51)^	The Netherlands/PHASE study	*n* 175; 12·1 ± 0·4 years; 49 % girls	PA	1 year	Accelerometer: LPA, MVPA (min/d) Global Positioning System (GPS) loggers and Geographical Information Systems (GIS) data: child’s LPA and MVPA at home, school, local sports grounds, shopping centres and other locations. Transport-related LPA and MVPA (min/d)	Changes mean (95 % CI): PA across locations/domains: During school time LPA –11·0 (–19·5, −2·6), after school time LPA –25·0 (−32·8, −17·3) and MVPA – 8·0 (−12·1, −3·9). All *P* < 0·05. No changes for before school time LPA and MVPA, during school time MVPA, and weekend days LPA and MVPA. Context-specific PA: Before school time: LPA at home –3·4 (–5·3, −1·5). During school time: LPA on school grounds –19·0 (–29·0, −9·5), LPA and MVPA in other locations 14·4 (8·2, 20·6) and 2·4 (0·8, 4·0). After school time: MVPA on school grounds –1·1 (−1·9, −0·2), LPA on sports grounds −3·5 (–7·1, −0·01), and LPA − 21·2 (−29·0, −13·4) and MVPA − 5·2 (−7·3, −3·1) in other locations. All *P* < 0·05. No changes on weekend days for all locations Transport-related PA: Before school time: LPA for active transport 6·3 (5·3, 7·4) and LPA for passive transport –0·4 (–0·7, −0·1). After school time: LPA for passive transport −1·7 (−2·9, −0·4). Weekend days: LPA for active transport –15·9 (−25·2, −6·5). All *P* < 0·05. No changes for LPA during school time and MVPA (Longitudinal multilevel linear mixed models)	80 %
Ridley *et al.* ^(45)^	Australia	*n* 99; in 7th grade; 100 % girls;	PA	1 year	PAQ-C questionnaire, 5-point Likert scale, children are classified as active with a score of 3 or higher	- PAQ-C score: 7th grade: 2·9 ± 0·7; 8th grade: 2·5 ± 0·8 (*P* < 0·0001) Individual PAQ-C components: - Lunch/recess: 3 (2, 4) to 2 (2, 2) (*P* < 0·0001) - No significant difference was found for PE, after school, evenings and weekends (Paired *t* test and Wilcoxon rank order)	20 %†
Shin *et al.* ^(44)^	Korea/KCYPS study	*n* 1947; 12 years; 48 % girls	PA	1 year	Perceived Physical Education Activity (PPEA) measured by one item: ‘how many hours did you spend on your physical activity from physical education in the las week?’ 5-point Likert scale (1 = none to 5 = over 4 h)	PPEA (mean ± sd) from 3·1 ± 1·1 to 3·3 ± 1·3. Correlation: 0·29 (Correlation analyses)	0 %†
Vanwolleghem *et al.* ^(37)^	Belgium/no study name	*n* 313; 11·0 ± 0·5 years; 49 % girls	PA	2 years	The Flemish Physical Activity questionnaire: transport to school and to leisure-time destinations	- Change active transport to school: 65·2 % to 65·5 %. X^2^ 9·561, *P* > 0·05 - Change active transport to leisure-time destinations: 93·9 % to 70·0 %. X^2^ 0·133, *P* < 0·05 (Chi-square test)	40 %
PA and SB							
Bradley *et al.* ^(53)^	USA/CHIC studies	4th/5th grade: *n* 586; 50 % girls; 83 % Caucasian, 21 % African-American and 6 % other (in 6th/7th grade an additional cohort was added to the study (=first grade middle school))	PA SB	1 year	Activity questionnaire, based on the Know Your Body Health Habits Survey, including the type and frequency of the activity children usually did the most; MET level assigned to each activity	- Percent of youth classified as sedentary increased from 39·5 to 42·1 % for female students and from 25·2 to 26·6 % for male students after the transition - Significant decrease in number of vigorous activities (5–8 METs) (Mantel–Haenszel chi-square tests)	0 %†
Corder *et al.* ^(54)^	UK/SPEEDY study	*n* 990; 10·3 ± 0·3 years; 58 % girls	PA SB	4 years	Accelerometer: SB, LPA, MPA, VPA and MVPA (min/d).	- SB increased by 10·6 (95 % CI 9·0, 12·2) min/d/year - LPA decreased by 9·8 (95 % CI –10·7, –8·9) min/d/year - MVPA decreased by 2·9 (95 % CI –3·5, –2·3) min/d/year (Three-level mixed effects linear regression)	80 %
Morton *et al.* ^(22)^	UK/SPEEDY study	*n* 325; 10·2 ± 0·3 years; 52 % girls; 97 %white	PA SB	4 years	Accelerometer: proportion of wear time during SB, LPA and MVPA	- During the school day, time spent SB (mean ± sd) increased from 272·9 ± 25·5 to 300·2 ± 29·9 min, time spent LPA decreased from 80·7 ± 16·3 to 64·7 ± 18·9 min and MVPA day decreased from 30·8 ± 10·8 to 27·4 ± 13·7 min - During lunchtime, there was no change in time spent SB, an decrease in LPA from 17·0 ± 3·5 to 11·3 ± 4·6 min and a decrease in MVPA from 10·6 ± 4·9 to 6·5 ± 4·7 min (No statistical analysis)	60 %
Jaakkola *et al.* ^(59)^	Finland, Finnish Schools on the Move project	*n* 336 students; 49 % girls; mean age of 12 ± 0·38 years	PA SB	1 year	Accelerometer: MVPA (min/d) and SB (%/d)	- MVPA (mean ± sd) in boys decreased from 61·7 ± 26·6 to 57·9 ± 6·4, in girls from 47·5 ± 17·4 to 41·6 ± 18·5 - Sedentary time (mean ± sd) in boys increased from 64·2 ± 6·9 to 66·9 ± 7·1, in girls from 66·4 ± 5·7 to 70·9 ± 6·1 (No statistical analysis)	40 %
Marks *et al.* ^(55)^	Australia/no study name	*n* 243; 12·2 years; 60 % girls; 85 % Australian born	PA SB	5–8 months	Accelerometer: mean time (min) spent in MVPA, LPA and SB, meeting PA recommendations Self-report behavioural questionnaires: PA, active transport and screen behaviour	- All students showed a decline in daily MVPA (mean ± sd)(–4 min ± 13), LPA (–23 min ± 33), cycle/scoot to/from school (–0·7 ± 4·1 times/week), and an increase in daily after-school being very active (10 min ± 66) All *P* < 0·05 - No changes in PA behaviour were found for mean daily weekend being very active and walk to/from school (0–10 times/week) - Increases in daily SB (mean ± sd)(16 min ± 76), weekday leisure screen time (17 min ± 126) and weekday homework screen time (25 min ± 67), all *P* < 0·05 - No changes in sedentary/screen behaviour were found for weekend leisure screen time (min/d) (Exact McNemar or Bowker paired test of proportions or paired *t* test of means)	Accelerometer 80 % Questionnaire 60 %
Okazaki *et al.* ^(64)^	Japan	*n* 55; 9·9 ± 0·3 years; 56 % girls	PA SB	5 years	Accelerometer: mean minutes of SB, LPA and MVPA per d	- Increase SB (min/d (se)) by 3·3 (1·48), *P* = 0·025 - Decrease MVPA (min/d (se)) by 2·3 (0·71), *P* = 0·002 - Decrease LPA (min/d (se)) by 1·0 (1·16), *P* = 0·370 (Linear mixed models)	80 %
Ridgers *et al*.^(56)^	Australia/CLAN and HEAPS studies	*n* 773; 10–12 years old	Physical activity SB	3 years	Accelerometer: mean minutes of SB, LPA, MPA and VPA per valid day	- During recess (boys): %SB B: 25·2, 95 % CI: 19·8, 30·6, %LPA B: –11·2, 95 % CI: –15·0, –7·4, %MPA B: –8·5, 95 % CI: –10·5, –6·5, %VPA B: –4·2, 95 % CI: –5·1, –3·3. All *P* < 0·001 - During recess (girls): no significant changes for %LPA, %SB B: 22·6, 95 % CI: 17·1, 28·1, %MPA B: –10·6, 95 % CI: –12·7, –8·6, %VPA B: –8·0 95 % CI: –9·0, –7·0. All *P* < 0·001 - During lunchtime (boys): %SB B: 23·1, 95 % CI: 19·1, 27·1, %LPA B: –9·2, 95 % CI: –11·7, –6·8, %MPA B: –8·5, 95 % CI: –9·6, –7·4, %VPA B: –4·5, 95 % CI: –5·4, –3·6. All *P* < 0·001 - During lunchtime (girls): no significant changes for %LPA, %SB B: 17·6, 95 % CI: 13·5, 21·7, %MPA B: –9·7, 95 % CI: –10·9, –8·4, %VPA B: –6·4, 95 % CI: –7·4, –5·4, All *P* < 0·001 (Multilevel analysis)	80 %
Rutten *et al.* ^(58)^	Belgium/no study name	*n* 472; 11·0 ± 0·4 years; 55 % girls	PA SB	2 years	PA: pedometer for 7 d, mean daily steps + self-reported with the PAQ-C questionnaire. SB: self-reported questionnaire.	- No change was found in mean steps per d (for all categories) - TPA (1–5) in boys with NW changed from (mean ± sd) 3·1 ± 0·6 to 2·8 ± 0·51, *P* = 0·000. For boys OW and girls OW and NW no changes were found - School-related PA (1–5) in boys NW changed from 3·9 ± 0·7 to 2·2 ± 0·7, *P* = 0·000. For boys OW and girls OW and NW no changes were found - Leisure time PA (1–5) in boys NW changed from 2·6 ± 0·7 to 2·5 ± 0·7 *P* = 0·002, and girls NW changed from 2·5 ± 0·6 to 2·3 ± 0·6 *P* = 0·018. For boys, OW and girls OW no changes were found - Screen-based SB (h/week) in boys NW changed from 21·6 ± 14·6 to 23·8 ± 13,0, *P* = 0·000. For boys OW and girls OW and NW no changes were found - Homework (h/week) boys OW changed from 8·2 ± 5·4 to 7·6 ± 4·2, *P* = 0·033. For boys NW and girls OW and NW no changes were found (ANOVA)	40 %
Rutten *et al.* ^(57)^	Belgium/no study name	*n* 472; 11·0 ± 0·4 years; 55 % girls	PA SB	2 years	PA: pedometer for 7 d and PAQ-C questionnaire (MVPA). Screen-based SB: questionnaire	The steps per d (mean ± sd) increased from 15 052 ± 4264 to 15 589 ± 5110. MVPA decreased from 3·0 ± 0·6 to 2·6 ± 0·5. Screen-based SB increased from 19·7 ± 12·9 to 22·6 ± 13·5 (No statistical analysis)	40 %
SB							
Atkin *et al.* ^(60)^	UK/SPEEDY study	T0 *n* 1512; 10·3 ± 0·3 years; 55 % girls T4 *n* 319; 14·3 ± 0·3 years; 52 % girls	SB	4 years	Accelerometer: h/week sedentary time Questionnaire: self-reported screen time h/week	Sedentary time (mean ± sd) increased from 34·9 ± 5·3 to 40·3 ± 5·3 (h/week) Self-reported screen time (median IQR) increased from 6·9 (2·9–14·8) to 15·1 (8·5–26·0) (h/week) (No statistical analyses)	Accelerometer 60 % Questionnaire 40 %
Dietary behaviour							
Lytle *et al.* ^(16)^	USA/CATCH study	*n* 291; 45 % girls; 92·8 % Caucasian	Dietary behaviour	3 years	Weekday 24-h recalls	Prevalence: - Consumption of breakfast decreased from 94·4 % to 85·2 %. Consumption of lunch decreased from 100 % to 98·3 %. Fruit and vegetable consumption decreased from 55·9 % (fruit) and 49·5 (vegetables) to 37·1 % (fruit) and 41·6 % (vegetables). Drinking milk decreased from 97·9 % to 90·1 %. Drinking fruit juice decreased from 47·4 % to 32·0 %. Drinking soft drink increased from 30·8 % to 57·1 %. Eating high-fat salty snacks decreased from 59·9 % to 46·7 %. All *P* < 0·05. No difference in consumption of dinner, snacks, fruit-flavoured beverages, high-fat sweet snacks and fast food Mean daily frequency (mean number of eating occasions): - Drinking milk decreased from 2·2 to 1·9. Drinking fruit juice decreased from 0·6 to 0·4. Drinking soft drinks increased from 0·4 to 0·8. Eating high-fat salty snacks decreased from 0·8 to 0·6. All *P* < 0·05. No difference in consumption of fruit-flavoured drink, snacks and high-fat sweet snacks (ANOVA)	60 %
Marks *et al.* ^(61)^	Australia/no study name	*n* 243; 12·2 years; 60 % girls; 85 % Australian-born	Dietary behaviour	5–8 months	Eat Well Be Active questionnaire: school day about quantity and total intake of various food and drink items	- The daily intake of non-core food items (–1·2; 95 % CI, –1·7 to –0·7; *P* < 0·001) and SSB (–0·3; 95 % CI, –0·5 to –0·1; *P* < 0·001) decreased. - The intake of school-day fruit (–0·2; 95 % CI, –0·4 to –0·1; P=.003) and school-day vegetables (–0·2; 95 % CI, –0·3 to –0·1; *P* < 0·001) decreased. - No difference in daily frequency of fruit consumption (Exact McNemar or Bowker paired test of proportions or paired *t* test of means)	60 %
Oza-Frank *et al.* ^(62)^	USA/ECLS-K study	*n* 7445; 50 % girls; 58·4 % White	Dietary behaviour	3 years	Food consumption questionnaire: containing 19 questions about food and beverages	Green salad consumption (*P* < 0·01) from 2·2 to 2·6 servings per week. Potatoes intake from 2·1 to 2·2 servings per week. Carrots intake (*P* < 0·01) from 2·8 to 1·9 servings per week. Other vegetables (*P* < 0·05) from 5·2 to 4·9 servings per week. Fruit (*P* < 0·05) from 7·7 to 7·2 servings per week. Fast food (*P* < 0·01) from 3·2 to 2·4 servings per week. Total servings of caloric beverages from 21·6 to 19·7 (*P* < 0·01). Drinking milk from 10·3 to 8·5 servings per week (*P* < 0·05). Servings of 100 % fruit juice from 5·1 to 5·6 servings per week (*P* < 0·01). (*t* tests)	20 %†
Ross *et al.* ^(63)^	USA/TRACK study	*n* 260; 10·5 ± 0·6 years; 59 % girls; 33 % White, 43 % Black, 9 % Hispanic and 15 % other race/ethnicity	Dietary behaviour	2 years	Total diet quality: 24-h recall (Block Food Screener)	Total diet quality declined –0·4 points per year (*P* < 0·001) (Growth curve analysis)	60 %
Winpenny *et al.* ^(15)^	UK/SPEEDY study	*n* 351; 10·2 ± 0·3 years; 56 % girls	Dietary behaviour	4 years	Food diaries: mean daily consumption of macronutrients (% en) and food groups	Mean change total daily intake of energy KJ b (se): 247 KJ (64) (*P* < 0·001), %En protein: 0·04 (0·20), %En carbohydrates: 0·43 (0·35), En% sugar: –2·64 (0·44) (*P* < 0·001), %En fat: –0·56 (0·33), %En sat. fat: –0·54 (0·18) (*P* < 0·01), NSP g/MJ: 0·07 (0·03) (*P* < 0·05), confectionary g/MJ: - 0·63 (se: 0·25) (*P* < 0·05), savoury snacks: 0·26 (0·14), SSB g/MJ: 4·66 (1·87) (*P* < 0·05), fruits g/MJ: –3·13 (1·14) (*P* < 0·01), vegetables g/MJ: 1·55 (0·48) (*P* < 0·01), fries g/MJ: 1·31 (0·39) (*P* < 0·01). (Multilevel mixed effects logistic and linear regression)	60 %
PA, SB and dietary behaviour							
Dowda *et al.* ^(36)^	USA/TRACK study	*n* 658; 11·0 ± 0·5 years; 55 % girls; 40 % white, 33 % Black, 9 % Hispanic and 18 % other race/ethnicity	PA, SB and diet quality	2 years	Accelerometer: MVPA (min/h) and SB (min/h) 24-h recalls (self-report) (one week): diet quality (41 specific food items, plus frequency and amount of intake) (higher score (max = 50) indicates better quality)	- MVPA (mean ± sd) declined (boys): from 3·7 ± 2·1 (5th grade) to 3·4 ± 1·8 (6th grade) min/h (NS) - SB increased significantly: from 31·0 ± 4·2 (5th grade) to 34·1 ± 4·1 (6th grade) min/h time - Diet quality decreased significantly From 29·8 ± 5·5 (5th grade) to 28·5 ± 6·0 (6th grade) - MVPA significantly declined (girls): from 2·2 ± 1·2 (5th grade) to 1·9 ± 1·0 (6th grade) min/h - SB increased significantly: from 32·8 ± 4·4 (5th grade) to 37·0 ± 4·5 (6th grade) min/h over time. - Diet quality decreased significantly from 30·3 ± 5·4 (5th grade) to 29·5 ± 5·6 (6th grade) (Chi-square and *t* tests)	40 %

PA, physical activity; PAQ-C, Physical Activity Questionnaire for Older Children; PE, physical education; PPEA, perceived physical education activity; LPA, light intensity physical activity; MVPA, moderate-to-vigorous physical activity; TPA, total physical activity; NW, normal weight; SB, sedentary behaviour; MPA, moderate physical activity; VPA, vigorous physical activity; OW, overweight; NSP, non-starch polysaccharide; SSB, sugar-sweetened beverages.

*Studies that produced multiple papers are listed together.

† Studies with a poor quality rating are not mentioned in the evidence synthesis.

## Results

### Search results

**Fig. 1:**
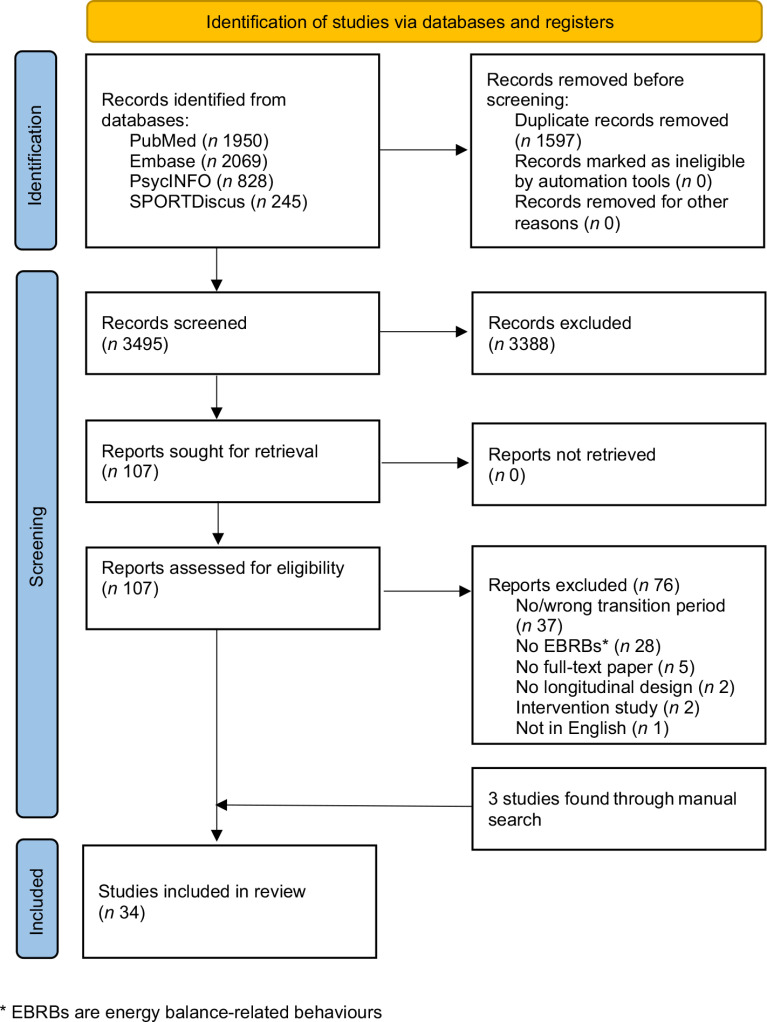
Flow chart of the review process

After removing duplicates, the search yielded 3495 unique hits (see Fig. 1). Screening of titles and abstracts resulted in 107 potentially eligible studies. Full-text screening resulted in thirty-one studies that met the inclusion criteria. Three additional studies were included after a manual search of reference lists. This resulted in the inclusion of total thirty-four studies.

### Study characteristics

Table 2 presents the characteristics of the included studies, which were conducted in the USA, Australia, UK, Belgium, Finland, Japan, Korea, the Netherlands and Norway. Eighteen studies focused on PA^(21,28,37–52)^, eight on PA and SB^(22,53–59)^, one on SB^(60)^, one on PA, SB and dietary intake^(36)^, five on dietary intake^(15,16,61–63)^ and none on sleep behaviour. In total, twenty-four studies used data from cohort studies, including the CHIC, PEACH, TRACK, SPEEDY, PASS, APPLES, CATCH, ECLS-K, CLAN, KCYPS and HEAPS studies. All studies were published between 1998 and 2021 with sample sizes ranging from 99 to 7445 participants. The average participant age at the time point at primary school ranged from 10 to 12 years and follow-up from 5 months to 4 years. Table 3 summarises nineteen strong and moderate quality studies reporting evidence on energy balance-related behaviours (i.e. PA, SB, sleep behaviour and dietary behaviour) across the transition from primary to secondary school.

**Table 3:** Summary of evidence on changes in energy balance-related behaviours across the school transition

Physical activity			Sedentary behaviour		Dietary behaviour
TPA	**−** ±^a^ ±^bc^ ±^d^	Sedentary behaviour	+ **+ + + +**	Fruit and vegetable	- - -
LPA	**0 - -±^b^ ±^d^**	Screen time	±^d^ ±^bc^	SSB	− + +
MVPA	**- - -** - **-** + + **±^bd^ ±^d^** ±^b^			Unhealthy snacks	− ±^e^ ±^e^
Active transport	**0** + +±^e^ **±^df^**			Fruit juice	–
Extracurricular PA	–			Milk	–
School related PA	±^bc^			Total energy intake	+
				Dietary fibre	+
				Intake from sugars	–
				Intake of SFA	–
				Energy percentage from protein	0
				Energy percentage from carbohydrates	0
				Energy percentage from fat	0
				Total diet quality	–

TPA, total physical activity; SSB, sugar-sweetened beverages; MVPA, moderate-to-vigorous physical activity; PA, physical activity.

Note that only strong and moderate quality studies were included in the evidence synthesis. In bold = study with high quality rating, + = a significant improvement in behaviour, − = significant worsening in behaviour, 0 = no change in behaviour, ± = inconsistent findings within a study, a = different results for different measurement method, b = different results for boys and girls, c = different results for different weight categories, d = different results for different time segments of the day or week, e = different results in subcategories of energy balance-related behaviours and f = different results for different intensity levels of active transport.

### Physical activity

PA was assessed using accelerometers^(21,22,28,36,38,40,41,43,46,47,49–52,54–56,59,64)^, self-report questionnaires^(21,28,37,38,42,44,45,48,50,53,55)^, pedometers^(57,58)^, parent-reported questionnaires^(50)^, activity logs^(39)^, Global Positioning System (GPS), loggers and Geographical Information Systems (GIS) data^(51)^. Fourteen studies examined multiple PA measures^(21,22,28,38,43,48,50,51,54–58,64)^ and fourteen studies examined one PA measure: TPA, MVPA, level of PA (duration and intensity of sixteen activities), active/non-active classification (active defined as having a score of at least 3 out of 5 points on the Physical Activity Questionnaire for Older Children), Perceived Physical Education Activity (PPEA), active transport and number of vigorous activities^(36,37,39–42,44–47,49,53,59)^. Eight studies received a strong methodological quality rating^(38,43,46,49,51,54,56,64)^. Three studies received a strong methodological quality rating for the accelerometer-based data and a moderate quality rating for the questionnaire-based data^(21,28,55)^. Twelve studies received a moderate quality rating^(22,36,37,40,41,47,48,50,52,57–59)^, and five studies received a poor quality rating^(39,42,44,45,53)^. Sixteen studies were included in the evidence synthesis^(21,28,36,37,40,43,45,46,49,51,52,54–56,58,64)^.

Seven studies examined the change in TPA across the transition from primary to secondary school. One study with moderate quality rating for the questionnaire-based data and strong quality rating for the accelerometer-based data showed a significant decrease in questionnaire-based TPA (min/d), but no change in accelerometer-based TPA or MVPA (min/d)^(21)^. Three strong quality studies based on the TRACK data showed a significant decrease in overall TPA (min/h)^(46,49)^, TPA during school time, TPA after school time and TPA during evening time^(43)^. One moderate quality study showed a significant decrease in TPA among boys with a healthy weight, but not among boys with overweight or girls with or without overweight^(58)^. The last moderate quality study examined TPA in various times of the day, and the study showed a significant decrease in the number of adolescents classified as active and decrease in TPA during recess and lunchtime, but no changes were found in physical education (PE), PA after school time, PA in the evenings and PA in the weekends^(45)^.

Five strong quality studies examined the change in LPA^(51,54–56,64)^, of which two studies showed a significant decrease in LPA (min/d)^(54,55)^, one study showed no changes^(64)^ and one study showed a significant decrease among boys but not among girls^(56)^. One study showed a significant decrease in LPA during and after school time (min/d), but no changes before school time and on weekend days. Context-specific results showed a decrease in LPA before school time at home, during school time at school, and after school time at sports grounds and other locations^(51)^. There was a significant increase in LPA during school time at other locations (e.g. at friend’s homes or at parks). No changes were found for weekend days^(51)^.

Ten studies examined the change in MVPA of which two studies examined moderate physical activity (MPA) and vigorous physical activity (VPA)^(21,28,36,43,51,52,54–56,64)^. We combined findings of the MPA and VPA studies with those of the studies examining MVPA, as results for these behaviours were in the same direction. Four studies with a strong quality rating showed a significant decrease in total MVPA^(54,55,64)^ and MVPA during recess and lunchtime^(56)^. Two moderate quality studies showed a significant decrease in MVPA of which one among girls only^(36,52)^. Two studies with strong quality rating showed a significant increase in weekday MVPA^(21,28)^. One study, with a strong quality rating, examined MVPA during various times in the day and showed a significant decrease in MVPA during school time among boys and girls, during the evening among girls only, but no changes in MVPA after school time^(43)^. The last study, with a strong quality rating showed a significant decrease in MVPA after school time, but no changes before school time, during school time and on weekend days^(51)^. Context-specific data showed a decrease in MVPA after school time at school and at other locations, a significant increase in MVPA during school time at other locations, while no changes were found before school time and on weekend days^(51)^.

One study with strong quality rating^(51)^ and four studies with a moderate quality rating examined the change in active transport^(21,37,40,55)^. The strong quality study showed a significant increase in active transport-related LPA before school time and a decrease in passive transport-related LPA before school time, after school time and during weekends, but no changes in active and passive transport-related MVPA^(51)^. Three moderate quality studies showed a significant increase in active transportation to/from school (min/d)^(21,37)^ or MVPA when commuting (min/d)^(40)^, of which one showed no change in active transport to leisure-time destinations^(37)^. The last study with moderate quality showed a significant decrease in times per week cycling or scooting to/from school, while no changes were found for walking to/from school^(55)^.

Two studies with a moderate quality rating did not fit the previous categories. One study found a significant decrease in extracurricular PA^(21)^. Another study found a significant decrease in school-related PA among boys with a healthy weight, and leisure-time PA among boys and girls with a healthy weight, but no changes among boys and girls with overweight^(58)^.

Overall, based on inconsistent findings, we found inconclusive evidence for a change in TPA, LPA, MVPA and active transport of adolescents across the transition from primary to secondary school.

### Sedentary behaviour

SB was assessed using accelerometers^(22,36,54–56,59,60,64)^ and questionnaires^(53,55,57,58,60)^. Three studies received a strong methodological quality rating^(54,56,64)^, and one study received a strong methodological quality rating for the accelerometer-based data and a moderate quality rating for the questionnaire-based outcomes^(55)^. Six studies received a moderate methodological quality rating^(22,36,57–60)^, and one study received a poor quality rating^(53)^. Six studies could be included in the evidence synthesis^(36,54–56,58,64)^.

Five studies examined the change in sedentary time, of which four studies showed a significant increase across the transition from primary to secondary school. Two of these studies received a strong quality rating^(54,64)^, one a strong quality rating for the accelerometer-based data^(55)^ and one a moderate quality rating^(36)^. One study with a strong quality rating found a significant increase in the proportion of sedentary time during recess and lunchtime^(56)^.

Two studies with a moderate quality rating examined the change in self-reported screen time across the school transition. One of the studies showed a significant increase in screen time for homework and leisure time during the week, but not during the weekend^(55)^. The other study showed a significant increase in screen time in boys with a healthy weight but no changes in girls or boys with overweight^(58)^.

Overall, we found strong evidence for an increase in SB of adolescents across the transition from primary to secondary school. We found inconclusive evidence for a change in screen time across the transition due to inconsistent results.

### Dietary behaviour

Dietary behaviours were assessed using 24-h recalls^(16,36)^, FFQ^(61–63)^ and food diaries^(15)^. Two studies examined overall diet quality^(36,63)^. Four studies examined the consumption of fruit, vegetable, snacks and SSB^(15,16,61,62)^. Three studies examined additional dietary behaviours, with one study examining breakfast, lunch, milk, and fruit-flavoured beverage consumption^(16)^, one study examining total energy intake and macro- and micronutrient intake^(15)^, and one study examining milk consumption^(62)^. Five studies received a moderate quality rating^(15,16,36,61,63)^ and one a poor quality rating^(62)^. Five studies were included in the evidence synthesis^(15,16,36,61,63)^.

Three studies with a moderate quality rating examined the change in fruit and vegetable consumption across the transition from primary to secondary school, all showing a significant decrease in consumption^(15,16,61)^.

Three studies with a moderate quality rating examined the change in unhealthy snack consumption^(15,16,61)^. One study showed a significant decrease in the consumption of non-core food items, such as potato chips and chocolate^(61)^. One study showed a significant increase in the consumption of fries and confectionary, but no change in the consumption of other savoury snacks^(15)^. The last study showed a significant decrease in the consumption of high-fat salty snacks, but no change in the consumption of overall snacks, and high-fat sweet snacks consumption^(16)^.

Three studies with a moderate quality rating examined the change in consumption of SSB^(15,16,61)^. Two studies showed a significant increase in the consumption of SSB^(15,16)^. One of these studies showed a significant decrease in the consumption of fruit juice^(16)^. A third study showed a significant decrease in the consumption of SSB^(61)^.

Three studies with a moderate quality rating did not fit the previous categories. Two studies based on data from the TRACK study showed a significant decrease in total diet quality^(36,63)^, and one study showed a significant decrease in the consumption of milk^(16)^. The last study showed a significant increase in total energy intake and dietary fibre intake and a significant decrease in total daily energy intake from sugars and the intake of SFA^(15)^. In this study, no significant changes were found for daily energy percentages from protein, carbohydrates and fat^(15)^.

Overall, we found moderate evidence for a decrease in fruit and vegetable consumption of adolescents across the primary to secondary school transition. Studies on unhealthy snack and SSB consumption showed inconsistent results leading to inconclusive evidence. The outcomes in studies that did not fit the previous categories were only reported once, leading to inconclusive evidence.

## Discussion

This systematic review summarised the evidence on changes in energy balance-related behaviours (i.e. PA, SB, sleep behaviour and dietary behaviour) of adolescents across the transition from primary to secondary school. We found strong evidence for an increase in SB, moderate evidence for a decrease in fruit and vegetable consumption, and inconclusive evidence for a change in TPA, LPA, MVPA, active transport, screen time, unhealthy snack and SSB consumption. No studies were identified examining the change in sleep behaviour across the transition from primary to secondary school.

Our results regarding inconclusive evidence for a change in TPA, LPA and MVPA across the transition from primary to secondary school is in contrast with previous literature. A review on PA change during adolescence (e.g. age-related literature not specifically focused on the school transition) found evidence for a decrease in PA (combining various outcomes of PA) in growing adolescents^(65)^. Another study found a decline in TPA and MVPA when adolescents grow older^(66)^. We found inconsistent results for a change in MVPA across the transition from primary to secondary school. Most of the included studies examining MVPA showed a significant decrease in total MVPA^(54,55)^, MVPA during recess and lunchtime^(56)^, and MVPA during school time but not after school time^(43)^. Remarkably, two studies showed a significant increase in weekday MVPA^(21,28)^. Our findings correspond to a recent review showing that changes in 24-h movement behaviours across the school transition largely depend on the time segments of the day or week^(13)^. The increase in weekday MVPA across the transition might be explained by an increase in active transport. Although we found inconclusive evidence for an increase in active transport in the current review, three out of five studies showed a significant increase in MVPA during commuting^(40)^ and active transportation to/from school^(21,37)^. One study found an increase in active transport-related LPA during weekdays and a decrease during weekend days across the school transition^(51)^. Generally, the distance to/from school increases as adolescents transition from primary to secondary school, which can result in an increase in active transport^(28)^. Conversely, an increased distance to/from school can also result in an increase in SB due to using passive, public transportation^(28)^.

The finding of an increase in SB across the transition from primary to secondary school is consistent with previous studies in adolescents that showed an increase in SB when adolescents grow older^(54,67)^, and with a review that found an increase in SB across the primary to secondary school transition^(14)^. However, our finding of inconclusive evidence for a change in screen time across the transition is in contrast to the findings of the review by Pearson *et al.* who showed an increase in screen time across the school transition^(14)^. Different inclusion criteria regarding the transition from primary to secondary school might explain this difference. In the present review, studies had to describe clearly that at least one measurement was taken in adolescents attending primary school and one in the same adolescents attending secondary school. Five studies included in the review of Pearson *et al.* did not meet our inclusion criterion because they did not mention a transition from primary to secondary school.

We found moderate evidence for a decrease in fruit and vegetable consumption and inconclusive evidence for a change in unhealthy snack and SSB consumption. This is partly confirmed in one cross-sectional study that found a decrease in fruit consumption and no change for vegetable consumption with increasing age^(68)^. A review including age-related studies found a lack of evidence for many potential determinants of fruit and vegetable consumption in children and adolescents, especially for determinants related to the physical and social school environment^(69)^. Studies on determinants of fruit and vegetable consumption across the transition from primary to secondary school are currently lacking. Based on previous studies, we expected a significant increase in unhealthy snack and SSB consumption due to adolescents experiencing more freedom and receiving more pocket money across the school transition from primary to secondary school^(25–27,70)^. An important remark regarding studies examining dietary behaviour is the use of many different self-report measures often of unknown validity and reliability^(71)^. Consequently, the studies included in the present review examining dietary behaviour received a low-quality rating resulting in inconclusive evidence.

No studies on sleep behaviour were available that met our inclusion criteria. However, as mentioned in the introduction, we do expect changes in sleep behaviour across the transition from primary to secondary school. A study in Australian children showed that the majority of 10–11-year-olds met the minimum sleep requirements on school nights (9–11 h), while a quarter of 12–13-year-olds did not meet the minimum sleep requirements on school nights (8–10 h)^(72)^. More research is needed to investigate sleep behaviour across the primary to secondary school transition.

The results from this review suggest a worsening in aspects of the energy balance-related behaviours PA, SB and dietary behaviour across the transition from primary to secondary school. Energy balance-related behaviours are connected and strengthen each other, for example, an increase in screen time has been associated with an increase in unhealthy snack consumption, a decrease in fruit and vegetable consumption^(73)^ and less sleep^(10)^. Interventions targeting these energy balance-related behaviours during the transition from primary to secondary school therefore seem warranted. In the current review, nine out of thirty-three included studies examined more than one behaviour, of which eight on PA and SB^(22,53–59)^, and one on PA, SB and dietary intake^(36)^. In these studies, the outcomes of these behaviours were linked as results indicate that PA decreases were often replaced by SB^(53–56)^. However, more longitudinal research is needed on changes in energy balance-related behaviour across the school transition, especially regarding sleep behaviour. Moreover, future research should focus on how energy balance-related behaviours influence each other in the school transition. Furthermore, qualitative research regarding the reasons for changes in behaviours related to the change in school environment is needed in order to develop appropriate interventions. To the best of our knowledge, current interventions do not specifically target the school transition period but mainly focus on primary or secondary school. Moreover, many school-based interventions targeting PA and dietary behaviour exist, while only a few target healthy sleep behaviour^(74)^.

Seven out of thirty-three studies included in the present review received a strong methodological quality rating^(38,43,46,49,52,54,56)^. Three studies received a strong methodological quality rating for the accelerometer-based data and a moderate quality rating for the questionnaire-based data^(21,28,55)^. Quality items that limited the methodological quality rating of a study included a follow-up rate below 70 %, not having a representative sample, or not adjusting for potential confounders in the statistical analysis. Future studies should keep these potential sources of bias in mind when designing their study in order to conduct high-quality studies.

### Strength and limitations

This review is the first summarising changes in dietary behaviour across the transition from primary to secondary school. Furthermore, this is the first review including all four energy balance-related behaviours (PA, SB, sleep behaviour and dietary behaviour) in a systematic review on changes in these behaviours across the school transition, which adds information to previous reviews by Pearson *et al.* and Chong *et al.* that only included two or three behaviours^(13,14)^. Other strengths of this review include the broad search strategy, which included four electronic databases without publication data restrictions. Furthermore, two independent reviewers conducted title and abstract screening, quality assessment, and data extraction resulting in the elimination of bias and errors in the methodology. A limitation is that we could have missed relevant studies that did not clearly state that the measurements were taken in adolescents attending primary school and in the same adolescents attending secondary school. We applied this strict inclusion criterion because we were interested in transitions accompanying a change in school environment, as such transitions may influence adolescents’ energy balance-related behaviours^(55)^. Another limitation is that only studies published in English were included. Additionally, we did not include grey literature in our search strategy. Furthermore, conducting a meta-analysis was not feasible because of the heterogeneity in outcomes and research methods in the included studies. In this review, we found inconsistencies between study results that are due to differences in measurement, setting and outcome. We recommend to develop and use an agreed set of key outcomes to be measured and reported in all future studies examining changes in energy balance-related behaviours to benefit evidence synthesis from all published studies^(75)^. Furthermore, we recommend future studies to provide more detailed characteristics of the school setting as a difference in setting could explain the different results between studies. For example, one study could have included schools that provided school meals, while another study included schools without school meals. This specific information about characteristic in the setting could not be extracted from the included studies. Lastly, the findings may not be generalisable to the adolescents of low- and middle-income countries because all studies were conducted in high-income countries.

## Conclusion

The current review found strong evidence for an increase in SB and moderate evidence for a decrease in fruit and vegetable consumption of adolescents across the transition from primary to secondary school. There was inconclusive evidence for the other energy balance-related behavioural outcomes due to inconsistent results and lack of high-quality studies. More longitudinal research is needed specifically on changes in energy balance-related behaviour across the school transition, especially regarding sleep behaviour. These studies should keep potential sources of bias in mind when designing their study in order to conduct high-quality studies.

## Supplementary material

For supplementary material accompanying this paper visit https://doi.org/10.1017/S1368980023000812. click here to view supplementary material
