# Supplementary material for: Is the transition from primary to secondary school a risk factor for energy balance-related behaviours? A systematic review
Source: Public Health Nutr. 2023 May 4;26(9):1754–74. doi: 10.1017/S1368980023000812 (PMC10478069; doi:10.1017/S1368980023000812)
Supplement: Supplementary file 1 [file S1368980023000812sup001.docx]

**Supplementary file 1.**

Search strategy PubMed

| Search | Query |
| --- | --- |
| #8 | (((#1 AND #2) AND (OR #3 OR #4 OR #5 OR #6 OR #7))) |
| #7 | ((energy balance-related behavi* [tiab] OR obesity-related behavi*[tiab])) |
| #6 | (("Sleep"[Mesh] OR sleep*[tiab] OR “time in bed”[tiab] OR “bed time”[tiab] OR bedtime[tiab] OR “night rest”[tiab] OR night awakening*[tiab] OR night waking*[tiab] OR “daytime sleepiness”[tiab] OR “day-time sleepiness”[tiab])) |
| #5 | (("Food"[Mesh] OR nutrition[tiab] OR food[tiab] OR diet[tiab] OR intake[tiab] OR beverages[tiab] OR “sugar sweetened beverages”[tiab] OR “energy drinks”[tiab] OR “energy drink”[tiab] OR carbonated beverage*[tiab] OR carbonated drink*[tiab] OR “fast foods”[tiab] OR “fast food”[tiab] OR candy[tiab] OR snack[tiab] OR snacking[tiab] OR snacks[tiab] OR “snack food”[tiab] OR “soft drink”[tiab] OR fruit[tiab] OR fruits[tiab] OR vegetables[tiab] OR vegetable[tiab] OR drinks[tiab] OR soda[tiab] OR drinking[tiab] OR breakfast[tiab] OR supper[tiab] OR lunch[tiab] OR meal[tiab] OR dinner[tiab] OR tea[tiab] OR fat[tiab] OR “5-a-day”[tiab] OR eating[tiab])) |
| #4 | (("exercise"[MeSH] OR “sports”[Mesh] OR Physical Activit*[tiab] OR exercis*[tiab] OR sport[tiab] OR sports[tiab] OR active[tiab] OR activities[tiab] OR activity[tiab] OR cycling[tiab] OR running[tiab] OR recreation[tiab] OR play[tiab] OR jogging[tiab] OR walk*[tiab] OR bicycle[tiab] OR cycle[tiab] OR bicycling[tiab] OR rowing[tiab] OR swim*[tiab])) |
| #3 | (("Sedentary Lifestyle"[Mesh] OR "computer time"[tiab] OR "computer use"[tiab] OR "gaming"[tiab] OR physical inactivit*[tiab] OR "prolonged sitting"[tiab] OR "screen time"[tiab] OR "screen-time"[tiab] OR sedent*[tiab] OR "sitting time"[tiab] OR stationary behavio*[tiab] OR "television"[tiab] OR "TV time"[tiab] OR "TV viewing"[tiab] OR "watching TV"[tiab] OR "cell phone"[tiab] OR “mobile phone”[tiab])) |
| #2 | ((“cohort study”[All Fields] OR cohort[All Fields] OR “cohort studies”[All Fields] OR “cohort analysis”[All Fields] OR “longitudinal study”[All Fields] OR longitudinal[All Fields] OR prospective[All Fields] OR “prospective study”[All Fields] OR “prospective studies”[All Fields] OR “follow up study”[All Fields] OR “follow up studies”[All Fields] OR “follow-up study”[All Fields] OR “follow-up studies”[All Fields] OR tracking[All Fields] OR “epidemiological method”[All Fields] OR “epidemiological methods”[All Fields] OR “epidemiological study”[All Fields] OR “epidemiological studies”[All Fields] OR “observational study”[All Fields] OR “observational studies”[All Fields] OR observational[All Fields] OR "Follow-up Studies"[MeSH Terms] OR "Prospective Studies"[MeSH Terms] OR "Longitudinal Studies"[MeSH Terms])) |
| #1 | ((((primary[tiab] OR middle[tiab] OR elementary[tiab]) AND (secondary[tiab] OR high[tiab])) OR ((primary[tiab] OR elementary[tiab]) AND (secondary[tiab] OR middle[tiab] OR high[tiab]))) AND school[tiab]) |

Search strategy Embase

| #8 | (#1 AND #7) AND (#2 OR #3 OR #4 OR #5 OR #6) |
| --- | --- |
| #7 | ‘cohort study’ OR ‘cohort’ OR ‘cohort studies’ OR ‘cohort analysis’ OR ‘longitudinal study’ OR ‘longitudinal’ OR ‘prospective’ OR ‘prospective study’ OR ‘prospective studies’ OR ‘follow up study’ OR ‘follow up studies’ OR ‘follow-up study’ OR ‘follow-up studies’ OR ‘tracking’ OR ‘epidemiological method’ OR ‘epidemiological methods’ OR ‘epidemiological study’ OR ‘epidemiological studies’ OR ‘observational study’ OR ‘observational studies’ OR ‘observational’ OR 'longitudinal study'/exp OR 'prospective study'/exp |
| #6 | ‘energy balance-related behavi*’:ab,ti OR ‘obesity-related behavi*’:ab,ti |
| #5 | 'sleep'/exp OR ‘sleep*’:ab,ti OR ‘time in bed’:ab,ti OR ‘bed time’:ab,ti OR ‘bedtime’:ab,ti OR ‘night rest’:ab,ti OR ‘night awakening*’:ab,ti OR ‘night waking*’:ab,ti OR ‘daytime sleepiness’:ab,ti OR ‘day-time sleepiness’:ab,ti |
| #4 | 'adolescent nutrition'/exp OR 'child nutrition'/de OR 'feeding behavior'/exp OR 'food'/de OR 'beverage'/exp OR 'fast food'/exp OR 'fat'/exp OR 'fruit'/exp OR 'health food'/exp OR 'sugar'/exp OR 'vegetable'/exp OR 'dietary intake'/exp OR ‘nutrition’:ab,ti OR ‘food’:ab,ti OR ‘diet’:ab,ti OR ‘intake’:ab,ti OR ‘beverages’:ab,ti OR ‘sugar sweetened beverages’:ab,ti OR ‘energy drinks’:ab,ti OR ‘energy drink’:ab,ti OR ‘carbonated beverage*’:ab,ti OR ‘carbonated drink*’:ab,ti OR ‘fruit vegetable juice’:ab,ti OR ‘fast foods’:ab,ti OR ‘fast food’:ab,ti OR ‘candy’:ab,ti OR ‘snack’:ab,ti OR ‘snacking’:ab,ti OR ‘snacks’:ab,ti OR ‘snack food’:ab,ti OR ‘soft drink’:ab,ti OR ‘fruit’:ab,ti OR ‘fruits’:ab,ti OR ‘vegetables’:ab,ti OR ‘vegetable’:ab,ti OR ‘drinks’:ab,ti OR ‘soda’:ab,ti OR ‘drinking’:ab,ti OR ‘breakfast’:ab,ti OR ‘supper’:ab,ti OR ‘lunch’:ab,ti OR ‘meal’:ab,ti OR ‘dinner’:ab,ti OR ‘tea’:ab,ti OR ‘fat’:ab,ti OR ‘5-a-day’:ab,ti OR ‘eating’:ab,ti |
| #3 | ‘physical activity’/exp OR ‘exercise’/exp OR ‘sport’/exp OR ‘physical activit*’:ab,ti OR ‘exercis*’:ab,ti OR ‘sport’:ab,ti OR ‘sports’:ab,ti OR ‘active’:ab,ti OR ‘activities’:ab,ti OR ‘activity’:ab,ti OR ‘cycling’:ab,ti OR ‘running’:ab,ti OR ‘recreation’:ab,ti OR ‘play’:ab,ti OR ‘jogging’:ab,ti OR ‘walk*’:ab,ti OR ‘bicycle’:ab,ti OR ‘cycle’:ab,ti OR ‘bicycling’:ab,ti OR ‘rowing’:ab,ti OR ‘swim*’:ab,ti |
| #2 | 'sedentary lifestyle'/exp OR 'computer time':ti,ab OR 'computer use':ti,ab OR 'gaming':ti,ab OR 'physical inactivit*':ti,ab OR 'prolonged sitting':ti,ab OR 'screen time':ti,ab OR 'screen-time':ti,ab OR 'sedent*':ti,ab OR 'sitting time':ti,ab OR 'stationary behavio*':ti,ab OR television:ti,ab OR 'TV time':ti,ab OR 'TV viewing':ti,ab OR 'watching TV':ti,ab OR ‘cell phone’:ti,ab OR ‘mobile phone’:ti,ab |
| #1 | (((‘primary’:ab,ti OR ‘middle’:ab,ti OR ‘elementary’:ab,ti) AND (‘secondary’:ab,ti OR ‘high’:ab,ti)) OR ((‘primary’:ab,ti OR ‘elementary’:ab,ti) AND (‘secondary’:ab,ti OR ‘middle’:ab,ti OR ‘high’:ab,ti))) AND ‘school’:ab,ti |

Search strategy PsycINFO

| #8 | (#1 AND #7) AND (#2 OR #3 OR #4 OR #5 OR #6) |
| --- | --- |
| #7 | (DE "Longitudinal Studies" OR DE "Prospective Studies" OR DE "Followup Studies") OR (“cohort study” OR “cohort” OR “cohort studies” OR “cohort analysis” OR “longitudinal study” OR “longitudinal” OR “prospective” OR “prospective study” OR “prospective studies” OR “follow up study” OR “follow up studies” OR “follow-up study” OR “follow-up studies” OR “tracking” OR “epidemiological method” OR “epidemiological methods” OR “epidemiological study” OR “epidemiological studies” OR “observational study” OR “observational studies” OR “observational”) |
| #6 | TI (“energy balance-related behavi*” OR “obesity-related behavi*”) OR AB (“energy balance-related behavi*” OR “obesity-related behavi*”) |
| #5 | (DE "Sleep" OR DE "Napping" OR DE "NREM Sleep" OR DE "REM Sleep" OR DE "Sleepiness") OR TI (“sleep*” OR “time in bed” OR “bed time” OR “bedtime” OR “night rest” OR “night awakening*” OR “night waking*” OR “daytime sleepiness” OR “day-time sleepiness”) OR AB (“sleep*” OR “time in bed” OR “bed time” OR “bedtime” OR “night rest” OR “night awakening*” OR “night waking*” OR “daytime sleepiness” OR “day-time sleepiness”) |
| #4 | (DE “Fast Food” OR DE “Food Intake” OR DE “Nutrition” OR DE “Energy Drink” OR DE “Beverages (Nonalcoholic)” OR DE “Food”) OR TI (“nutrition” OR “food” OR “diet” OR “intake” OR “beverages” OR “sugar sweetened beverages” OR “energy drinks” OR “energy drink” OR “carbonated beverage*” OR “carbonated drink*” OR “fruit vegetable juice” OR “fast foods” OR “fast food” OR “candy” OR “snack” OR “snacking” OR “snacks” OR “snack food” OR “soft drink” OR “fruit” OR “fruits” OR “vegetables” OR “vegetable” OR “drinks” OR “soda” OR “drinking” OR “breakfast” OR “supper” OR “lunch” OR “meal” OR “dinner” OR “tea” OR “fat” OR “5-a-day” OR “eating”) OR AB (“nutrition” OR “food” OR “diet” OR “intake” OR “beverages” OR “sugar sweetened beverages” OR “energy drinks” OR “energy drink” OR “carbonated beverage*” OR “carbonated drink*” OR “fruit vegetable juice” OR “fast foods” OR “fast food” OR “candy” OR “snack” OR “snacking” OR “snacks” OR “snack food” OR “soft drink” OR “fruit” OR “fruits” OR “vegetables” OR “vegetable” OR “drinks” OR “soda” OR “drinking” OR “breakfast” OR “supper” OR “lunch” OR “meal” OR “dinner” OR “tea” OR “fat” OR “5-a-day” OR “eating”) |
| #3 | (DE "Exercise" OR DE "Aerobic Exercise" OR DE "Weightlifting" OR DE "Yoga" OR DE "Physical Activity" OR DE "Sports" OR DE "Baseball" OR DE "Basketball" OR DE "Extreme Sports" OR DE "Football" OR DE "Judo" OR DE "Martial Arts" OR DE "Soccer" OR DE "Swimming" OR DE "Tennis" OR DE "Weightlifting") OR TI (“physical activit*” OR “exercis*” OR “sport” OR “sports” OR “active” OR “activities” OR “activity” OR “cycling” OR “running” OR “recreation” OR “play” OR “jogging” OR “walk*” OR “bicycle” OR “cycle” OR “bicycling” OR “rowing” OR “swim*”) OR AB (“physical activit*” OR “exercis*” OR “sport” OR “sports” OR “active” OR “activities” OR “activity” OR “cycling” OR “running” OR “recreation” OR “play” OR “jogging” OR “walk*” OR “bicycle” OR “cycle” OR “bicycling” OR “rowing” OR “swim*”) |
| #2 | (DE "SEDENTARY behavior" OR DE "SEDENTARY lifestyles" OR DE "SEDENTARY behavior in children") OR TI (“computer time” OR “computer use” OR “gaming” OR “physical inactivit*” OR “prolonged sitting” OR “screen time” OR “screen-time” OR “sedent*” OR “sitting time” OR “stationary behavio*” OR television OR “TV time” OR “TV viewing” OR “watching TV” OR “cell phone” OR “mobile phone”) OR AB (“computer time” OR “computer use” OR “gaming” OR “physical inactivit*” OR “prolonged sitting” OR “screen time” OR “screen-time” OR “sedent*” OR “sitting time” OR “stationary behavio*” OR television OR “TV time” OR “TV viewing” OR “watching TV” OR “cell phone” OR “mobile phone”) |
| #1 | TI ((((“primary” OR “middle” OR “elementary”) AND (“secondary” OR “high”)) OR ((“primary” OR “elementary”) AND (“secondary” OR “middle” OR “high”))) AND “school”) OR AB ((((“primary” OR “middle” OR “elementary”) AND (“secondary” OR “high”)) OR ((“primary” OR “elementary”) AND (“secondary” OR “middle” OR “high”))) AND “school”) |

Search Strategy SPORTdiscus

| #8 | (#1 AND #7) AND (#2 OR #3 OR #4 OR #5 OR #6) |
| --- | --- |
| #7 | “cohort study” OR “cohort” OR “cohort studies” OR “cohort analysis” OR “longitudinal study” OR “longitudinal” OR “prospective” OR “prospective study” OR “prospective studies” OR “follow up study” OR “follow up studies” OR “follow-up study” OR “follow-up studies” OR “tracking” OR “epidemiological method” OR “epidemiological methods” OR “epidemiological study” OR “epidemiological studies” OR “observational study” OR “observational studies” OR “observational” |
| #6 | TI (“energy balance-related behavi*” OR “obesity-related behavi*”) OR AB (“energy balance-related behavi*” OR “obesity-related behavi*”) |
| #5 | (DE "SLEEP" OR DE "SLEEP hygiene" OR DE "SLEEP & health") OR TI (“sleep*” OR “time in bed” OR “bed time” OR “bedtime” OR “night rest” OR “night awakening*” OR “night waking*” OR “daytime sleepiness” OR “day-time sleepiness”) OR AB (“sleep*” OR “time in bed” OR “bed time” OR “bedtime” OR “night rest” OR “night awakening*” OR “night waking*” OR “daytime sleepiness” OR “day-time sleepiness”) |
| #4 | (DE "FOOD" OR DE "BEVERAGES" OR DE "FATS & oils" OR DE "FRIED food" OR DE "FRUIT" OR DE "JUNK food" OR DE "NATURAL foods" OR DE "PACKAGED foods" OR DE "PROCESSED foods" OR DE "SNACK foods" OR DE "SUGAR" OR DE "VEGETABLES" OR DE "NUTRITION" OR DE "CHILD nutrition" OR DE "FRUIT in human nutrition" OR DE "SNACK foods & health" OR DE "SUGARS in human nutrition" OR DE "VEGETABLES in human nutrition" OR DE "YOUTH -- Nutrition" OR DE "FOOD habits") OR TI (“nutrition” OR “food” OR “diet” OR “intake” OR “beverages” OR “sugar sweetened beverages” OR “energy drinks” OR “energy drink” OR “carbonated beverage*” OR “carbonated drink*” OR “fruit vegetable juice” OR “fast foods” OR “fast food” OR “candy” OR “snack” OR “snacking” OR “snacks” OR “snack food” OR “soft drink” OR “fruit” OR “fruits” OR “vegetables” OR “vegetable” OR “drinks” OR “soda” OR “drinking” OR “breakfast” OR “supper” OR “lunch” OR “meal” OR “dinner” OR “tea” OR “fat” OR “5-a-day” OR “eating”) OR AB (“nutrition” OR “food” OR “diet” OR “intake” OR “beverages” OR “sugar sweetened beverages” OR “energy drinks” OR “energy drink” OR “carbonated beverage*” OR “carbonated drink*” OR “fruit vegetable juice” OR “fast foods” OR “fast food” OR “candy” OR “snack” OR “snacking” OR “snacks” OR “snack food” OR “soft drink” OR “fruit” OR “fruits” OR “vegetables” OR “vegetable” OR “drinks” OR “soda” OR “drinking” OR “breakfast” OR “supper” OR “lunch” OR “meal” OR “dinner” OR “tea” OR “fat” OR “5-a-day” OR “eating”) |
| #3 | (DE "EXERCISE" OR DE "ABDOMINAL exercises" OR DE "AEROBIC exercises" OR DE "ANAEROBIC exercises" OR DE "ARM exercises" OR DE "BACK exercises" OR DE "CHAIR exercises" OR DE "CHEST exercises" OR DE "CIRCUIT training" OR DE "EXERCISE for children" OR DE "EXERCISE for girls" OR DE "EXERCISE for youth" OR DE "EXERCISE video games" OR DE "FOOT exercises" OR DE "GYMNASTICS" OR DE "KNEE exercises" OR DE "LEG exercises" OR DE "MUSCLE strength" OR DE "PILATES method" OR DE "RUNNING" OR DE "RUNNING -- Social aspects" OR DE "SCHOOL exercises & recreations" OR DE "SHOULDER exercises" OR DE "STRENGTH training" OR DE "TREADMILL exercise" OR DE "YOGA" OR DE "PHYSICAL activity" OR DE "PHYSICAL training & conditioning" OR DE "SPORTS" OR DE "PLAY") OR TI (“physical activit*” OR “exercis*” OR “sport” OR “sports” OR “active” OR “activities” OR “activity” OR “cycling” OR “running” OR “recreation” OR “play” OR “jogging” OR “walk*” OR “bicycle” OR “cycle” OR “bicycling” OR “rowing” OR “swim*”) OR AB (“physical activit*” OR “exercis*” OR “sport” OR “sports” OR “active” OR “activities” OR “activity” OR “cycling” OR “running” OR “recreation” OR “play” OR “jogging” OR “walk*” OR “bicycle” OR “cycle” OR “bicycling” OR “rowing” OR “swim*”) |
| #2 | (DE "SEDENTARY behavior" OR DE "SEDENTARY lifestyles" OR DE "SEDENTARY behavior in children") OR TI (“computer time” OR “computer use” OR “gaming” OR “physical inactivit*” OR “prolonged sitting” OR “screen time” OR “screen-time” OR “sedent*” OR “sitting time” OR “stationary behavio*” OR television OR “TV time” OR “TV viewing” OR “watching TV” OR “cell phone” OR “mobile phone”) OR AB (“computer time” OR “computer use” OR “gaming” OR “physical inactivit*” OR “prolonged sitting” OR “screen time” OR “screen-time” OR “sedent*” OR “sitting time” OR “stationary behavio*” OR television OR “TV time” OR “TV viewing” OR “watching TV” OR “cell phone” OR “mobile phone”) |
| #1 | TI ((((“primary” OR “middle” OR “elementary”) AND (“secondary” OR “high”)) OR ((“primary” OR “elementary”) AND (“secondary” OR “middle” OR “high”))) AND “school”) OR AB ((((“primary” OR “middle” OR “elementary”) AND (“secondary” OR “high”)) OR ((“primary” OR “elementary”) AND (“secondary” OR “middle” OR “high”))) AND “school”) |

**Supplementary file 2.** Quality assessment per included study, sorted by energy balance-related behaviour, study name, quality score and alphabetically by first author.

| **Author (year)** | **Research question** | **Study population** | **Representative sample** | **Recruitment of subjects** | **Sample size** | **Outcome measures** | **Follow up rate** | **Statistical analysis** | **Quality assessment** |
| --- | --- | --- | --- | --- | --- | --- | --- | --- | --- |
| **Physical activity** | | | | | | | | | |
| **Clennin et al. (49)** | + | + | + | + | + | + | + | + | 100% |
| **Dowda et al. (48)** | + | + | + | + | - | - | + | - | 60% |
| **Dowda et al. (50)** | + | + | + | + | - | Parent reported child’s PA: -  Self-reported PA and accelerometer-based MVPA: + | - | - | Parent reported child’s PA: 40%  Self-reported PA and accelerometer-based MVPA: 60% |
| **Lau et al. (43)** | + | - | CD | + | - | + | + | + | 80% |
| **Pate et al. (46)** | + | + | + | CD | - | + | + | + | 80% |
| **Pate et al. (47)** | + | + | + | CD | - | + | - | - | 40% |
| **Pate et al.** |  |  |  |  |  |  |  |  |  |
| **Coombes et al. (38)** | + | + | + | + | - | + | + | - | 80% |
| **Cooper et al. (28)** | + | + | + | + | - | Accelerometer: + Questionnaire: CD | + |  | Accelerometer 80%  Questionnaire 60% |
| **Jago et al. (41)** | + | + | - | + | - | + | + | - | 60% |
| **De Meester et al. (21)** | + | + | + | + | - | Accelerometer: + Questionnaire: - | - | + | Accelerometer 80%  Questionnaire 60% |
| **Garcia et al. (39)** | + | - | CD | CD | - | - | - | - | 0%^a^ |
| **Harrison et al. (40)** | + | + | - | + | - | + | - | - | 40% |
| **Kirby et al. (42)** | + | + | CD | + | - | - | - | - | 20%^a^ |
| **Mikalsen et al. (52)** | + | + | CD | + | + | + | - | + | 60% |
| **Remmers et al. (51)** | + | + | - | + | - | + | + | + | 80% |
| **Ridley et al. (45)** | + | + | - | + | + | - | - | - | 20%^a^ |
| **Shin et al. (44)** | + | - | CD | CD | - | NR | CD | - | 0%^a^ |
| **Vanwolleghem et al. (37)** | + | + | - | + | - | - | - | - | 40% |
| **Physical activity and Sedentary behaviour** | | | | | | | | | |
| **Bradley et al. (53)** | + | - | CD | CD | - | - | CD | - | 0%^a^ |
| **Corder et al. (54)** | + | + | + | + | - | + | - | + | 80% |
| **Morton et al. (22)** | + | + | + | + | - | + | - | - | 60% |
| **Jaakkola et al. (59)** | + | + | + | CD | - | + | - | - | 40% |
| **Marks et al. (55)** | + | + | - | + | - | Accelerometer: +  Questionnaire: - | + | + | Accelerometer 80%  Questionnaire 60% |
| **Okazaki et al. (64)** | + | + | + | - | - | + | + | + | 80% |
| **Ridgers et al. (56)** | + | + | - | + | - | + | + | + | 80% |
| **Rutten et al. (58)** | + | + | - | + | - | - | - | + | 40% |
| **Rutten et al. (57)** | + | + | + | + | - | - | - | - | 40% |
| **Sedentary behaviour** | | | | | | | | | |
| **Atkin et al. (60)** | + | + | + | + | - | Accelerometer: +  Questionnaire: - | - | - | Accelerometer 60%  Questionnaire 40% |
| **Dietary behaviour** | | | | | | | | | |
| **Lytle et al. (16)** | + | + | + | - | - | - | + | + | 60% |
| **Marks et al. (61)** | + | + | - | + | + | - | + | + | 60% |
| **Oza-Frank et al. (62)** | + | + | CD | + | - | CD | CD | - | 20%^a^ |
| **Ross et al. (63)** | + | + | + | + | - | - | - | + | 60% |
| **Winpenny et al. (15)** | + | + | + | + | - | - | - | + | 60% |
| **Physical activity, sedentary behaviour, and dietary behaviour** | | | | | | | | | |
| **Dowda et al. (36)** | + | - | CD | CD | - | Accelerometer: +  Questionnaire: + | - | - | 40% |
| **Sleep behaviour** | | | | | | | | | |
| **Meltzer et al. (75)** |  |  |  |  |  |  |  |  |  |

^a = studies with a poor quality rating are not mentioned in the evidence synthesis, + = Yes, - = No, CD = Cannot Determine, NA = Not Applicable, NR = Not Reported, * = validity/precision criteria^
